# Pyrethroid Resistance in an *Anopheles funestus* Population from Uganda

**DOI:** 10.1371/journal.pone.0011872

**Published:** 2010-07-29

**Authors:** John C. Morgan, Helen Irving, Loyce M. Okedi, Andrew Steven, Charles S. Wondji

**Affiliations:** 1 Liverpool School of Tropical Medicine, Liverpool, United Kingdom; 2 National Livestock Resources Research Institute, Tororo, Uganda; BMSI-A*STAR, Singapore

## Abstract

**Background:**

The susceptibility status of *Anopheles funestus* to insecticides remains largely unknown in most parts of Africa because of the difficulty in rearing field-caught mosquitoes of this malaria vector. Here we report the susceptibility status of the *An. funestus* population from Tororo district in Uganda and a preliminary characterisation of the putative resistance mechanisms involved.

**Methodology/Principal Findings:**

A new forced egg laying technique used in this study significantly increased the numbers of field-caught females laying eggs and generated more than 4000 F_1_ adults. WHO bioassays indicated that *An. funestus* in Tororo is resistant to pyrethroids (62% mortality after 1 h exposure to 0.75% permethrin and 28% mortality to 0.05% deltamethrin). Suspected DDT resistance was also observed with 82% mortality. However this population is fully susceptible to bendiocarb (carbamate), malathion (organophosphate) and dieldrin with 100% mortality observed after exposure to each of these insecticides. Sequencing of a fragment of the sodium channel gene containing the 1014 codon conferring pyrethroid/DDT resistance in *An. gambiae* did not detect the L1014F *kdr* mutation but a correlation between haplotypes and resistance phenotype was observed indicating that mutations in other exons may be conferring the knockdown resistance in this species. Biochemical assays suggest that resistance in this population is mediated by metabolic resistance with elevated level of GSTs, P450s and pNPA compared to a susceptible strain of *Anopheles gambiae*. RT-PCR further confirmed the involvement of P450s with a 12-fold over-expression of *CYP6P9b* in the Tororo population compared to the fully susceptible laboratory colony FANG.

**Conclusion:**

This study represents the first report of pyrethroid/DDT resistance in *An. funestus* from East Africa. With resistance already reported in southern and West Africa, this indicates that resistance in *An. funestus* may be more widespread than previously assumed and therefore this should be taken into account for the implementation and management of vector control programs in Africa.

## Introduction

Malaria is the main cause of morbidity and mortality in Uganda with around 12 million annual cases treated in the public health system alone (Malaria control program, Ministry of Health). Malaria control in Uganda relies heavily on vector control through the use of insecticide treated nets (ITNs), long-lasting insecticide nets (LLINs) and indoor residual spraying (IRS) mostly in regions of seasonal transmission. The success of such interventions requires a good knowledge of vector populations particularly their susceptibility status to the main insecticides used for such control programmes in order to detect and monitor resistance to these insecticides. The two major causes of insecticide resistance are alterations in the target sites and increases in the rate of insecticide metabolism. Three enzyme families, the cytochrome P450s, the esterases and glutathione S-transferases (GSTs), are primarily responsible for metabolic resistance [1].

The main malaria vectors in Uganda are *An. gambiae* and *An. funestus*, with *An. arabiensis* also involved [2]. Recent study showed that *An. funestus* as *An. gambiae* was an omnipresent vector in Uganda, both representing around 87 and 100% of the collected anopheline mosquitoes depending of the area [2].

In Uganda, resistance to pyrethroids and DDT is mainly reported in *An. gambiae* [3], [4] and there are no published studies on insecticide resistance in *An. funestus* from this country, mainly because of difficulty in rearing field-caught mosquitoes of this vector with larvae difficult to find and adults laying fewer eggs in the laboratory compared to a species such as *An. gambiae*. Information on the susceptibility status of populations of this major malaria vector is crucial to successfully implement and manage present and future vector control programs. Indeed high level of insecticide resistance in this vector has already been associated in South Africa with an increase in malaria cases [5] highlighting the need to assess the susceptibility of populations of this species. So far cases of pyrethroid resistance in *An. funestus* have been reported only in Ghana, West Africa [6] and in southern Africa [7], [8]. However, no information is available for other parts of Africa notably East Africa.

We therefore undertook a study on the insecticide susceptibility status of *An. funestus* from Tororo district in Eastern Uganda. A preliminary characterisation of the major resistance mechanisms was also conducted.

## Materials and Methods

### Area of study and mosquito collection

Blood fed *An. funestus* adult females resting indoor were collected in houses between 06 and 12 AM in Tororo District (0°45′ N, 34°5′E) in Eastern Uganda, an area of high malaria transmission [2]. The collection was carried out in April and November 2009. Blood-fed and gravid mosquitoes resting inside houses were collected using aspirators and torches and immediately transported to the laboratory of the National Livestock Resources Research Institute based in Tororo. A new method was used to induce the females to lay eggs. Briefly, the collected blood fed females were stored in net covered paper cups or in cages and provided with cotton wool moistened with sucrose. They were maintained for 4 to 5 days to allow them to fully reach the gravid stage and were checked daily for survival. The gravid mosquitoes were then gently individually introduced into 1.5 ml Eppendorf tubes containing a 1square cm filter paper inserted into the bottom of the tube. The filter paper was moistened and excess water removed. The cap of the Eppendorf tube was pierced with 3 holes to allow air into the tube. The tubes were checked daily for the presence of eggs. Females that laid eggs were carefully removed from the tubes and transferred into Eppendorf tubes with silica gel. Eggs were stored at room temperature or at 4°C for up to 2 days and then sent via courier to the Liverpool School of Tropical Medicine (LSTM) where they were allowed to hatch in small cup and later transferred to larvae bowls for rearing. Apart from 20 families that were reared individually, the egg batches were pooled and reared together. Larvae were comparatively reared in mineral (bottled) and distilled water and fed abundantly with TetraminTM baby fish food every day. Water of each larvae bowl was changed every two days to reduce the mortality. The F_1_ adults generated were randomly mixed in cages for subsequent experiments.

### *PCR-species* identification

All females used for individual oviposition were morphologically identified as belonging to the funestus group according to the key of [9]. A PCR was carried out using the protocol of [10] to confirm that all females that laid eggs were *An. funestus s.s*.

### Insecticide susceptibility assays

Insecticide susceptibility assays were carried out using 2–5 day-old F_1_ adults from both individual and pooled families following the WHO protocol [11]. Around 20–25 mosquitoes per tube were exposed to insecticide-impregnated filter paper for 1 h and then transferred to a clean holding tube supplied with 10% sugar and mortality was determined 24 h later. We tested the following insecticides: the pyrethroids permethrin (0.75%), and deltamethrin (0.05%); the carbamate bendiocarb (0.01); the organophosphate malathion (5%) and the organochlorines DDT (4%) and dieldrin (4%).

### PBO synergist study

As P450 monooxygenases have previously been involved in pyrethroid resistance in *An. funestus* [12], [13] their potential involvement in the resistance was assessed in the Tororo population using PBO (piperonyl butoxide), an inhibitor of P450s activity [14]. The synergist PBO effect was analysed in combination with 0.75% permethrin. 100 female mosquitoes were pre-exposed to 4% PBO paper for 1 h and immediately exposed to 0.75% permethrin for 1 h. Final mortality was assessed after 24 h and compared to the results obtained without PBO.

### Biochemical assay

Biochemical assays based on the methods described by Penilla et al (1998) [15] were carried out using 50 female adults aged between 1 to 3 days from the Tororo mixed F_1_ mosquito sample. The *An. gambiae* kisumu strain [16] was used as the susceptible control sample since no susceptible strain from *An. funestus* was available, as done previously by Casimiro et al (2006, 2007) [8], [17]. The following enzyme assays were carried out: glutathione-S-transferase (GST), altered acetylcholinesterase (*AChE*), esterase assays (pNPA rate reaction, α and β esterases) and monooxygenases (P450s). We used a two-sample t-test to compare the results of the biochemical assays between the susceptible strain (Kisumu) and the field samples from Tororo following an adjustment for total protein content.

### Sodium channel gene sequencing for detection of *kdr* mutation

A fragment of the sodium channel gene spanning the 1014 codon associated with knockdown resistance (*kdr*) in *An. gambiae* [18], [19] was amplified and sequenced in pyrethroid/DDT resistant and susceptible mosquitoes from Tororo in order to detect any mutation associated with pyrethroid/DDT resistance. DNA was extracted using the LIVAK method [20] and amplified using primers Kdrfun-F GTT CAA TGA AGC CCC TCA AA and Kdrfun-R CCG AAA TTT GAC AAA AGC AAA. The PCR was carried out using 10 pmol of each primers and 30 ng of genomic DNA as template in 25 µl reactions containing 1X Kapa Taq buffer, 0.2 mM dNTPs, 1.5 mM MgCl_2_, 1U Kapa Taq (Kapa biosystems). The cycle parameters were: 1 cycle at 95°C for 5 min; 35 cycles of 94°C for 30 s, 57°C for 30 s and elongation at 72°C for 1 min; followed by 1 cycle at 72°C for 10 min. Sequences were aligned using ClustalW [21] while haplotypes reconstruction and polymorphism analysis were done using DnaSP v5.0 [22].

### Transcription profiling of candidate P450 genes

We carried out a RT-PCR analysis of mosquitoes for each copy of two duplicated P450 genes (*CYP6P9a, CYP6P9b, CYP6P4a, CYP6P4b*) previously found to be associated with pyrethroid resistance in a laboratory resistant strain originating from southern Mozambique (FUMOZ-R) [13] in order to see if they were also over-expressed in the Ugandan field samples. The Superscript III kit (Invitrogen) was used to assess the expression ratio of each gene relative to the ribosomal SP7 (*RSP7*) housekeeping gene. RNA was extracted using the Picopure RNA isolation kit (Arcturus) from three batches of 10 permethrin resistant females and 3 batches of 10 permethrin resistant males from the *An. funestus* population of Tororo. The same was done for the susceptible laboratory (FANG) strain. The primers used are presented in Table 1. *RSP7* primers were designed using the *An. gambiae* sequence AGAP010592-RA. The RT-PCR was carried out in duplex (candidate gene *+ RSP7*) using 10 pmol of each primers and 25 ng RNA as template in 15 µl reactions containing 2X reaction buffer, 1U Superscript enzyme mix (SSIII Platinum Tag Mix). The cycle parameters were: 1 cycle at 55°C for 30 min and 94°C for 2 min followed by 30 cycles of 94°C for 15 s, 58°C for 30 s and elongation at 68°C for 1 min; followed by 1 cycle at 68°C for 5 min. The relative expression of each gene was quantified using the GeneTools software from the SynGene gel picture system and normalised against the *RSP7* expression.

**Table 1:** Primers used for the RT-PCR.

Genes	Forward sequence (5′-3′)	Reverse sequence (5′-3′)	Expected size
***CYP6P9a***	ATGTGATAAACG AAACACTTCGCAA common to both CYP6P9 copies and spanning the unique intron	TTTATTATAGATTGGAAG TATCTCA	453 bp
***CYP6P9b***		TACAAAAACCCCTTCCGC TGCACC	471 bp
***CYP6P4a***	ATGTGATCAACGAAA CTCTACGCAAAT (common to both CYP6P4 copies and spanning the unique intron	CGTTTCCATGGAATTACA TTTTCTG	503 bp
***CYP6P4b***		ACAATCATTATACCACAC ATCTGAC	502 bp
***RSP7***	AGAACCAGCAGACCA CCATC	GCTGCAAACTTCGGCTATTC	186 bp

## Results

### Mosquito collection

A total of 1500 indoor-resting *Anopheles* mosquitoes were collected inside houses in Tororo over a 2 week period in April and another two weeks in November 2009. Around 700 were morphologically identified as belonging to the *An. funestus* group, 750 were identified as belonging to the *An. gambiae* complex and the remaining 50 to other Anopheles species.

### Mosquito rearing

From the 300 oviposition Eppendorf tubes set up with individual gravid *An. funestus* females, around 230 egg batches were obtained for combined April and November collections. These eggs were transported to Liverpool where they were successfully reared to the adult stage. Larvae reared in mineral (bottled) water grew quicker and look fitter than those reared in distilled water as seen recently for the rearing of *An. funestus* larvae from Mozambique [23]. The rearing time from egg to adult was 16 days and more than 4000 F_1_ adult mosquitoes were obtained. The results from the PCR-species identification confirmed that all the females that laid eggs were *An. funestus* s.s.

### Susceptibility tests

Bioassays carried out using randomly mixed F_1_ individuals generated from the 230 egg batches, indicated that the *An. funestus* population from Tororo was resistant to both type I and type II pyrethroids. Indeed, a mortality rate of 62% was recorded when female *An. funestus* were exposed to 0.75% permethrin, type I pyrethroid, while a mortality rate of 79% was observed for males (Table 2). A higher resistance level was observed after 1 h exposure to 0.05% deltamethrin (type II pyrethroid) with a mortality of only 28% observed for females and 65% for males.

**Table 2:** WHO susceptibility test results on 2–5 day old F_1_ An. funestus from Tororo.

	Females		Males		Total	
	n	% mortality	n	% mortality	n	% mortality
Permethrin (0.75%)	577	62±3.6	394	79±4.1	971	69±3.7
Deltamethrin (0.05%)	137	28±4.4	52	65±5.7	189	39±4.9
DDT (4%)	137	82±5.6	53	89±6.5	190	83.6±6.1
Bendiocarb (0.01%)	85	100±0	50	100±0	135	100±0
Dieldrin (4%)	50	100±0	nd	nd	50	100±0
Malathion (5%)	50	100±0	nd	nd	50	100±0
PBO+Permethrin (0.75%)	101	90±3.2	nd	nd	101	90±3.2

nd; not done

A suspected resistance to DDT was also observed in the Tororo population of *An. funestus* but at a lower level than for pyrethroids, with a mortality rate of 82% for females and 89% for males after 1 h exposure to 4% DDT (Table 2).

No resistance was observed for bendiocarb (carbamate), malathion (organophosphate) and dieldrin (organochlorine) with 100% mortality at 1 h exposure for respectively 0.01% bendiocarb, 5% malathion and 4% dieldrin (Table 2).

A mortality of 90% was observed when 100 females were pre-exposed to 4% PBO and later immediately exposed to 0.75% permethrin indicating that P450 monooxygenases are playing a major role in the pyrethroid resistance mechanism of this Tororo *An. funestus* population.

### Biochemical assay results

**Figure 1:**
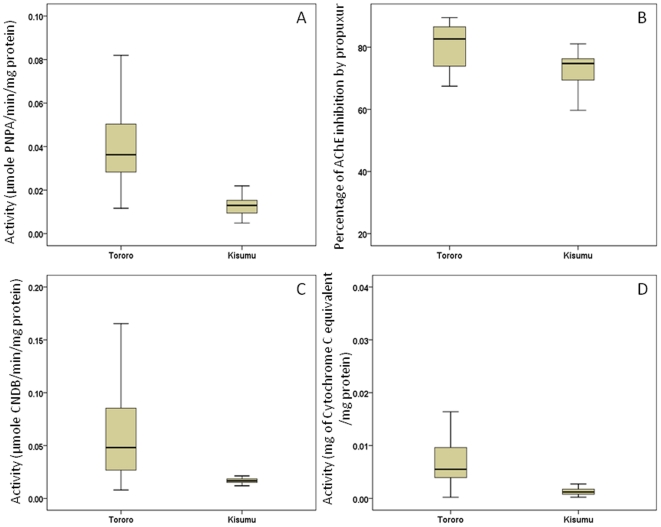
Box plots of results from biochemical assays. The median activity of the *An. funestus* population of Tororo compared with the *An. gambiae* Kisumu reference strain is shown by a horizontal bar; the box denotes the upper and lower quartiles. The vertical lines show the full range of the data set; (A) Range of esterase activity with the substrate *p*-nitrophenyl acetate; (B) Acetylcholinesterase inhibition ranges. (C); Range of GST activity. (D) Estimated levels of cytochrome P450s (representing monooxygenase activity).

A moderately significant increase in esterase activity was observed with the substrate pNPA in the Tororo population compared to the Kisumu susceptible strain (P = 0.031) (Figure 1A, Table 3). No significant difference was observed with either α and β-Naphthyl acetate substrates or in the percentage of *AChE* inhibition by propuxur (Figure 1B, Table 3).

**Table 3:** Comparisons of average values for a range of biochemical assays between F_1_ adult progeny *An. funestus* from Tororo populations and the *An. gambiae* Kisumu insecticide-susceptible reference strain.

	Mean Tororo	Mean Kisumu	Fold change	P value
pNPA	0.036	0.0126	2.8	0.031
α-Naphthyl acetate	8.33 10^−05^	3.68 10^−05^		
β-Naphthyl acetate	1.75 10^−05^	5.03 10^−05^		
P450	0.0094	0.004	2.35	0.02
GST	0.056	0.0161	3.47	0.001
AChE	78.2	72.4		

A significant increase in the level of GST activity was observed in the Tororo population compared to the susceptible Kisumu strain with mean CDNB levels 3.47-fold higher in the Tororo population (P<0.001) (Figure 1C, Table 3).

Increased level of monooxygenases (2.35 fold) were also detected in the Tororo population compared to the Kisumu strain (P<0.05) (Figure 1D, Table 3). These results suggest that a metabolic resistance mechanism is operating in the Tororo population.

### Transcription profiling of candidate P450 genes

**Figure 2:**
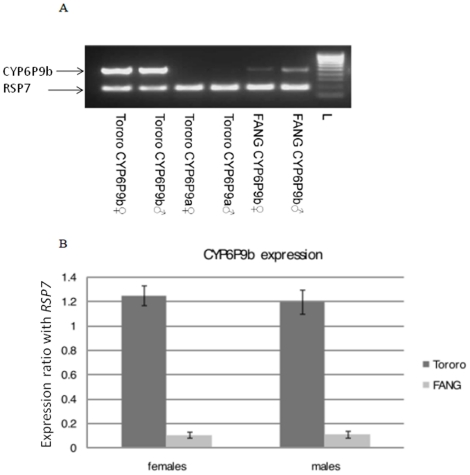
Transcription profile of candidate genes in the Tororo population. A) RT-PCR gel of both copies of *CYP6P9*. B) Comparison of the patterns of gene expression of *CYP6P9b* between the resistant field sample of Tororo and the laboratory susceptible strain FANG. The normalised expression ratio of *CYP6P9b* against *RSP7* gene is represented on the vertical axis.

The 2.35-fold increased in the level of monooxygenases observed above led us to analyse the Tororo population for the expression levels of two duplicated P450 genes recently associated with pyrethroid resistance in FUMOZ-R, a pyrethroid resistant laboratory strain [13]. The semi-quantitative amplification of the two copies of *CYP6P9* and *CYP6P4* showed that only *CYP6P9b*, was over-expressed in the Tororo population for both males and females (Figure 2A). *CYP6P4b* was not expressed but amplified from genomic DNA while despite several attempts and change of primers *CYP6P9a* as well as *CYP6P4a* was never amplified even from genomic DNA suggesting that copies of these genes may not be present in Tororo population. Overall, the *CYP6P9b* copy is 12 times over-expressed in females from Tororo compared to females of the susceptible FANG strain and 11-times for the males (Figure 2B).

### Sequencing of a fragment of sodium channel gene spanning the 1014 codon

**Figure 3:**
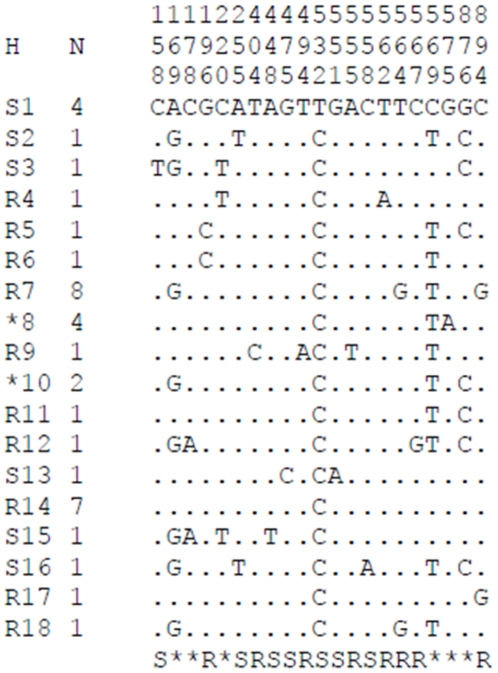
Schematic representation of haplotypes (H) of the 917 bp portion of the Voltage-gated sodium channel gene (VGSC) observed in the Tororo population. Only polymorphic sites are shown and these are numbered from the beginning of the 917 bp sequence. Dots mean identity with the first sequence. A number has been given to each haplotype preceded by the letter R or S if it is unique to the resistant or susceptible sample respectively. In case of a shared haplotype, the number is preceded by an asterisk. The column (N) indicates the number of individuals sharing the haplotype. Below the list of haplotypes, R or S indicates the positions that are polymorphic in the resistant or susceptible mosquitoes respectively, while an asterisk marks a position polymorphic in both phenotypes.

We amplified and sequenced a 994 bp fragment of the voltage-gated sodium channel (VGSC) gene. A total of nineteen Tororo *An. funestus* specimens were sequenced (5 DDT resistant mosquitoes, 8 permethrin resistant and 6 susceptible mosquitoes). A 917 bp sequence from this fragment was aligned between all individuals spanning 649 bp from intron 19, the entire exon 20 containing the 1014 codon (210 bp) and 59 bp in intron 20. The summary of the polymorphism of this fragment is presented in Table 4. Twenty one polymorphic sites were observed with 7 transitions and 14 transversions. No polymorphic site was observed in exon 20 meaning that no mutation was observed at the 1014 codon often associated with resistance in other insects. The amino acid sequence of exon 20 was 100% identical to that previously reported in other *An. funestus* samples [6], [24]. 18 haplotypes were observed in total (8 for susceptible and 12 for resistant, with 2 haplotypes in common) (Figure 3) and have been submitted to Genbank (Accession number HM193720-HM193437).

**Table 4:** Summary statistics for polymorphism at the sodium channel gene in susceptible and resistant *An. funestus* from Tororo, Uganda.

Samples	N	H	S	Ts	Tv	Single-tons	F	h	π (k)
**Susceptible**	12	8	13	7	6	6	33.3% (S1)	8 (0.894)	0.0043 (3.939)
**Resistant**	26	12	14	5	9	7	30.7% (R7)	12 (0.84)	0.0033 (3.025)
**Total**	38	18	21	7	14	10	21% (R7)	18 (0.912)	0.00377 (3.455)

N, number of sequences (2n); H, number of haplotypes (with 2 haplotypes in common); S, number of polymorphic sites; Ts, transition substitution; Tv, transversion substitution; F, frequency of the most common haplotype; h, Number of haplotypes (haplotype diversity); π, nucleotide diversity (k = mean number of nucleotide differences).

**Figure 4:**
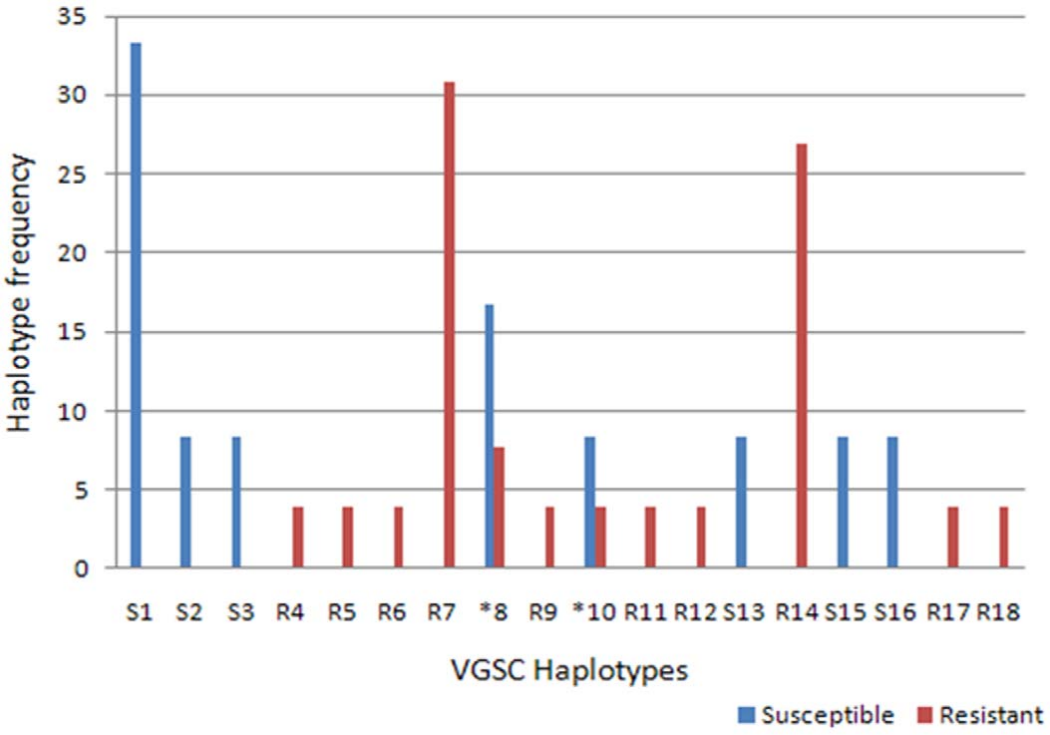
Distribution of the sodium channel gene haplotypes between susceptible and resistant mosquitoes from Tororo.

However, despite the lack of polymorphism in Exon 20, there are some indications of a correlation between the haplotypes and the resistance phenotype. Firstly, lower nucleotide (π) and haplotype diversities (h) were observed for resistant mosquitoes despite having a bigger sample size (0.0033 vs 0.0043 and 0.84 vs 0.894 for π and h respectively). Secondly, a higher proportion of singleton haplotypes is observed in susceptible (50%) than resistant 35% despite the bigger sample size for resistant mosquitoes. Thirdly, only 2 haplotypes out of 18 are shared between resistant and susceptible mosquitoes with the most common haplotypes for resistant and susceptible not share at all between the two phenotypes. Overall, there are two haplotypes (R7 and R14) shared by 57% of resistant individuals not present in the susceptible ones while there is one haplotype (S1) shared by 33% of susceptible individuals that is not represented in resistant ones (Figure 4). This analysis suggests a reduced genetic diversity in resistant mosquitoes potentially due to a selection associated with a mutation conferring resistance.

## Discussion

In this study, we assessed the susceptibility of an *An. funestus* population from Eastern Uganda against different insecticides and investigated the potential resistance mechanisms. This study has been significantly facilitated by the forced-egg laying method to generate high number of field mosquitoes for this species. *An. funestus* larvae are generally difficult to rear and therefore collection of field mosquitoes mostly relies on collecting blood-fed or gravid indoor resting females that are left to lay eggs. But quite often egg production from these field caught females is very low and limits the amount of bioassays that could be carried out to assess the susceptibility of populations of this malaria vector to insecticides or to carry out other genetic studies. The new method of forced-egg laying described here has been very successful in generating abundant F_1_ adult mosquitoes and has made possible the extensive assessment of the susceptibility status of this population against several insecticides. We hypothesise that the success of this method could be due to the stress caused to gravid female mosquitoes by the confinement in the tiny space of 1.5 ml tubes. Because this method of forced-egg laying is easy to implement, this represents a significant progress in working on field samples of this important malaria vector and should contribute to easily characterise this vector across Africa as it is done for field populations of *An. gambiae*. However, for studies carried out using such F_1_ adults to be informative, it is important that the F_1_ adults generated by this method are representative of the general population. For this purpose, the bias due to family effects should be minimised. To achieve that in this study, we pooled the egg batches and reared them together and the F_1_ adults were also randomly mixed in cages for subsequent experiments such as bioassays and biochemical assays. Samples for sequencing were taken from those alive or dead after bioassays. Therefore, we are confident that the potential impact of family effects was minimal on our results. This is further supported by the similarity of the bioassays results we obtained for both April and November collections. Analysis carried out by [25] using a non-linear regression model to infer genetic diversity estimates in large natural populations (such as *An. funestus*) from finite sample sizes (such as field collected females) have shown that a sample size of N = 50 is sufficient to capture most of the genetic diversity of the population and with egg batches from more than 200 females, the sample size of this study adequately reflect the *An. funestus* population of Tororo. Therefore, we will recommend using egg batches from around 50 randomly field-caught females to indeed reduce the family effect on results from F_1_ adults.

Resistance to both types I and II of pyrethroids was observed in *An. funestus* population of Tororo. It is the first time that such resistance is reported in an *An. funestus* population from East Africa. So far pyrethroid resistance in *An. funestus* was reported in populations from southern Africa in Mozambique and South Africa [5], [7], [8] and in Ghana in West Africa [6]. The observation of this resistance in other region of Africa may indicate that more *An. funestus* populations are resistant to pyrethroids than previously thought. Indeed, this species was so far considered as been mainly susceptible with the exception of few areas [26]. Probably the difficulty in obtaining large sample size of this mosquito may have prevented to assess the extent of the resistance problem in this species.

The pattern of pyrethroid resistance in *An. funestus* could be different to that of *An. gambiae* since we observed a higher frequency of deltamethrin resistance (type II pyrethroid) than against permethrin (type I pyrethroid) which is the contrary observed in *An. gambiae* populations of the same area [3]. Such higher frequency of deltamethrin resistance in *An. funestus* has been also observed in Mozambique [23]. This difference between the two species may be due to the underlying mechanisms of resistance which is mainly metabolic for *An. funestus* while *An. gambiae* exhibits both target-site (kdr) and metabolic resistance.

Moderate resistance to DDT was also observed in Tororo population of *An. funestus*. It is not the first case of multiple resistances in a population of *An. funestus*. Such multiple resistances have been already observed in *An. funestus* populations from Mozambique and Ghana [6], [8] with pyrethroid/carbamate resistance in Mozambique and pyrethroid/DDT/carbamate in Ghana. It remains to be established if cross-resistance mechanisms could be acting in these populations and could explain the multiple resistance observed. It has been suggested that a cross-resistance mechanism was acting in Mozambique to confer resistance to pyrethroids and carbamates through P450 mono-oxygenases [7]. Cross resistance between DDT and pyrethroids is often mainly conferred by kdr mutations in other mosquitoes such as *An. gambiae* or *Cx quinquefasciatus* [18], [19]. No mutations at the kdr locus were detected in *An. funestus* in Uganda. However, a correlation was observed between the haplotypes of a fragment of the sodium channel gene and the resistance phenotype. This is an indication that although no mutation has been found at the 1014 codon in exon 20, it is likely that one or more mutations located in other exons of the Voltage-gated sodium channel (VGSC) gene may be conferring the knockdown resistance in the *An. funestus* population of Tororo. Therefore, more exons should be sequenced particularly those where other mutations have previously been associated with knockdown resistance in other insects such as the exons 19 and 21 [27]. Another difference observed between resistant and susceptible samples was that most polymorphic sites present only in resistant mosquitoes were heterozygotes suggesting that if a *kdr* mutation was present in this population, it may still be predominantly heterozygote. However, more individuals should also been sequenced to confirmed the correlation observed in this study.

PBO synergistic study as well as results from biochemical assays and RT-PCR all indicate that resistance to pyrethroids and DDT observed in Tororo is mainly conferred by a metabolic resistance mechanism with P450 monooxygenases probably playing a major role as seen already in Mozambique [7], [8]. But the fact that 10% still survived after PBO pre-exposure indicates that other gene families than P450 monooxygenases may also be also involved. The elevated GST activity observed in Tororo compared to the susceptible sample suggests that GSTs are one of these other gene families involved in this resistance. They could particularly be active against DDT as shown in *An. gambiae* where *GSTe2* has been shown to be the main DDT metaboliser [20]. The absence of *CYP6P9* and *CYP6P4* duplication in *An. funestus* population of Tororo suggests a difference in the resistance mechanisms with the population from Mozambique where these two P450 genes are both duplicated and associated with pyrethroid resistance. Such difference highlights the need to analyse *An. funestus* populations across the whole Africa in order to fully characterise mechanisms conferring these resistances.

The source of selection responsible of this pyrethroid/DDT resistance remains to be elucidated. However because high level of resistance is also observed in *An. gambiae* from the same area [3], it is possible that the resistance exhibited by *An. funestus* has a common origin to that seen in *An. gambiae*.

### Conclusion

The detection of resistance to pyrethroids and DDT in *An. funestus* from Tororo calls for further studies to be carried out to establish the geographic distribution of this resistance across Africa and an assessment of its impact on malaria control programs.
